# Measuring and Predicting Sensor Performance for Camouflage Detection in Multispectral Imagery

**DOI:** 10.3390/s23198025

**Published:** 2023-09-22

**Authors:** Tobias Hupel, Peter Stütz

**Affiliations:** Institute of Flight Systems, University of the Bundeswehr Munich, 85577 Neubiberg, Germany; peter.stuetz@unibw.de

**Keywords:** multispectral, infrared, camouflage detection, target visibility, sensor performance, sensor management, performance modelling

## Abstract

To improve the management of multispectral sensor systems on small reconnaissance drones, this paper proposes an approach to predict the performance of a sensor band with respect to its ability to expose camouflaged targets under a given environmental context. As a reference for sensor performance, a new metric is introduced that quantifies the visibility of camouflaged targets in a particular sensor band: the Target Visibility Index (TVI). For the sensor performance prediction, several machine learning models are trained to learn the relationship between the TVI for a specific sensor band and an environmental context state extracted from the visual band by multiple image descriptors. Using a predicted measure of performance, the sensor bands are ranked according to their significance. For the training and evaluation of the performance prediction approach, a dataset featuring 853 multispectral captures and numerous camouflaged targets in different environments was created and has been made publicly available for download. The results show that the proposed approach can successfully determine the most informative sensor bands in most cases. Therefore, this performance prediction approach has great potential to improve camouflage detection performance in real-world reconnaissance scenarios by increasing the utility of each sensor band and reducing the associated workload of complex multispectral sensor systems.

## 1. Introduction

Multispectral sensor systems have become quite popular for various remote sensing applications, ranging from precision agriculture [1,2,3], land cover classification [4,5], detection of weeds [6], and plant disease monitoring [7,8,9] to shoreline extraction [10], water body detection [11], bathymetry [12], and disaster evaluation [13]. Their relatively low cost, size, weight, and power consumption make them suitable for use even on small reconnaissance drones, where the rich spectral information they provide can be utilized to detect camouflaged targets [14]. However, compared to the visual or thermal infrared sensors commonly used in reconnaissance scenarios, multispectral sensors provide a much larger number of bands, including derivatives such as vegetation indices (e.g., NDVI and NDRE). This additional information introduces a substantially heavier workload that must be managed by a sensor operator and possibly by any subsequent computer-aided processing system. For this reason, the Institute of Flight Systems at the University of the Bundeswehr, Munich, Germany is actively researching the use of multispectral sensor systems on small tactical drones in military reconnaissance scenarios.

Because each material has unique spectral characteristics, sensor bands that expose camouflaged targets in one environment, such as grassland, may not expose camouflaged targets in another environment, such as gravel. Knowing when to utilize which sensor band under given environmental conditions is usually based on experience and empirical experimentation. The large number of possible bands provided by multispectral sensor systems makes the selection of the most useful sensor bands an even more complex task, especially in time-critical military reconnaissance scenarios. Therefore, this work presents an approach to address this issue by predicting the performance of a sensor band with respect to its ability to expose camouflaged targets. More specifically, each sensor band is linked to a performance model that predicts its performance by assessing the current environmental situation. Having a measure of performance for each sensor band of a multispectral sensor system at flight time, the sensor bands can be ranked from those providing the most performance to those providing the least performance. Moreover, the sensor bands can be reduced to the most meaningful ones, leaving the sensor operator or any subsequent processing instance with a mere subset of all sensor bands. This subset is processed more quickly and is more likely to contain the information needed to detect camouflaged targets.

In order to quantify the performance of a sensor band, this work introduces the Target Visibility Index (TVI). The TVI is a metric that provides a measure of the extent to which a given sensor band exposes a camouflaged target. Using the TVI as a reference for sensor performance, machine learning models can be trained to learn the relationship between the current environmental situation and the corresponding performance of a given sensor band. After training, these machine learning models can be employed as performance models for the sensor bands of a multispectral sensor system, where they dynamically assess the environmental situation and predict the performance of their associated sensor band. Here, the environmental situation is represented in an abstract way by a so-called context state. The context state is a feature vector extracted by multiple feature descriptors from a preselected sensor band of the multispectral sensor system. In conclusion, the performance models technically learn the relationship between the context state and the TVI of their associated sensor band.

For the training of the performance models and the evaluation of their predictions, a custom dataset featuring 853 multispectral captures containing several different camouflaged targets in various environments at different seasons was compiled. To support reproducibility and enable further research, the dataset has been made publicly available for download (see the Data Availability Statement at the end of this manuscript).

In summary, this work makes the following scientific contributions:

- Proposition and evaluation of a method for predicting sensor performance with respect to the exposure of camouflaged targets.
- Introduction of a metric for measuring sensor performance with respect to the exposure of camouflaged targets.
- Provision of an extensive multispectral dataset containing multiple camouflaged targets: the eXtended Multispectral Dataset for Camouflage Detection (MUDCAD-X).

### 1.1. Related Work

Although research related to sensor performance modeling and prediction is scarce, there have been a number of recently conducted relevant studies. In [15], sensor performance models were used to map selected environmental states to the detection performance of object detection algorithms for flight trajectory optimization using an optimal control approach. Incorporating these sensor performance models into the optimization procedure allowed the computation of flight trajectories that maximized the detection performance of the object detection algorithms. In [16], object detection models were used to support the sensor scheduling algorithm by predicting the probability of successful object detections given the current environmental and UAV conditions for UAV-based multi-object tracking applications with limited sensor capabilities, leading to significantly improved object observation times. In other research, the most suitable detection algorithm has been dynamically selected aboard a sensor-equipped UAV under given environmental conditions through modelling and predicting the performance of several object detection algorithms using Bayesian Networks [17] or artificial neural networks and fuzzy inference [18]. Both of these approaches were able to substantially increase overall object detection performance. However, the prediction of sensor performance with respect to the exposure of camouflaged targets has not yet been explored, motivating the work presented in this manuscript.

The measurement of visibility or exposure of targets in a dynamic environment is a highly active field of research, especially in the automotive area, where traffic lights and signs have to be designed in such way that they cannot be overlooked by any road user. Visibility metrics based on luminance measurements and psychological behavior, such as the target visibility level [19] and the relative visual performance [20], have been proposed and evaluated in various scenarios [21,22,23,24]. The determination of visibility in terms of the distance at which objects can be identified from visual [25,26] and near-infrared [25] camera footage has been studied as well. For detecting the most salient regions and objects in an image according to human perception, a number of approaches have been introduced [27,28,29]. These methods generate a saliency map from an input image, highlighting those regions that the human eye would naturally focus on first. However, visibility metrics based on human perception in real-world scenes or laboratory environments are not applicable to the use case considered in this work, nor are visibility metrics in the form of viewing distances. Furthermore, saliency maps are expensive to compute and difficult to translate into a single sensor performance score. Therefore, in this paper we introduce a computationally inexpensive metric based on contrast [30] and statistical properties that have already been used for other image metrics [31,32].

### 1.2. Outline

In the following section (Section 2), the dataset, Target Visibility Index, and sensor performance prediction approach are introduced and explained in detail. The next section (Section 3) covers the evaluation and comparison of the machine learning models and their different training procedures with respect to their ability to determine the most informative bands given the context state. Finally, the results and their significance are discussed in Section 4, and summarized conclusions are drawn in Section 5.

## 2. Methods and Materials

This section introduces the dataset used to train and evaluate the proposed sensor performance prediction method in Section 2.1, the metric for target visibility in Section 2.2, and the proposed method itself in Section 2.3.

### 2.1. Dataset

**Figure 1:**
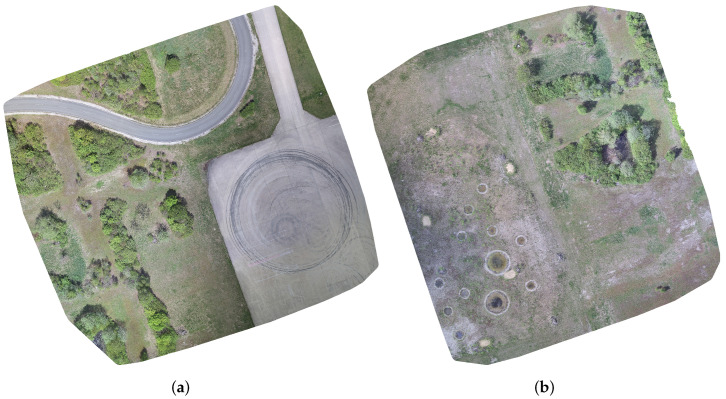
The two areas of the test site at the University of the Bundeswehr Munich (in May) where the camouflaged targets were placed: (**a**) area A; (**b**) area B.

**Figure 2:**
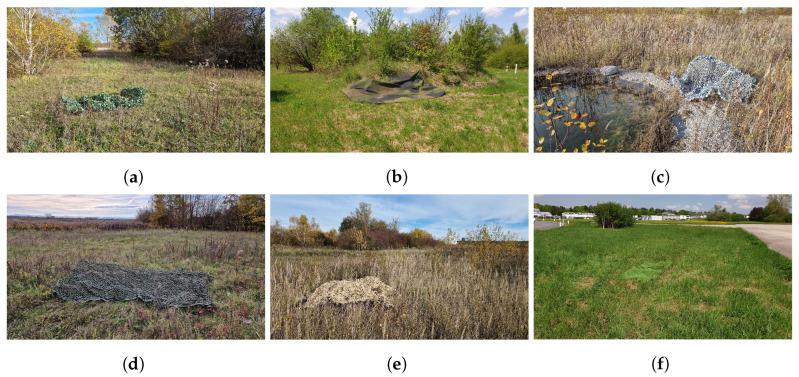
Multiple different camouflaged targets of the dataset from the ground perspective; all targets were placed in environments where they easily integrate: (**a**) artificial hedge, (**b**) green 2D camouflage net, (**c**) gray 3D camouflage net, (**d**) green 3D camouflage net, (**e**) yellow 3D camouflage net, (**f**) artificial turf.

The data used to train and evaluate the proposed sensor performance prediction approach were collected in two different areas of the test site at the University of the Bundeswehr, Munich. The areas shown in Figure 1 provided a variety of different environments, such as grassland, gravel and graveled soil, various bushes and trees, and both concrete and asphalt roads. This diversity constitutes an excellent foundation for a rich and comprehensive dataset. For the camouflaged targets, thirteen different objects were placed in visually similar environments in each of these areas: a piece of artificial turf, an artificial hedge, a green tarp, a green 2D camouflage net, a green 3D camouflage net, a gray tarp, an anthracite fleece, a gray 3D camouflage net, a yellow 3D camouflage net, and four persons, two wearing green uniforms and two wearing yellow uniforms. All targets are listed in Table 1 along with the corresponding target group indicating the type of environment in which the target was placed (i.e., green targets in green environments). In addition, the table shows the percentage distribution of the targets and target groups. As can be seen, the green targets dominate the data, making up almost two thirds, while the gray and yellow targets occupy about one and two ninths, respectively. This is due to the greater number of green targets compared to the number of yellow and gray targets as well as to the nature of the captured areas, which are dominated by green environments. Figure 2 shows the artificial hedge, the green 2D camouflage net, the gray 3D camouflage net, the green 3D camouflage net, the yellow 3D camouflage net, and the artificial turf in their corresponding environments from the ground perspective. Note that 3D camouflage nets have a more irregularly structured surface than 2D camouflage nets, which are mostly flat and similar to a tarp.

For acquisition of the multispectral data, the camouflaged targets were placed in one of the areas and captured from the nadir perspective by an unmanned aerial vehicle (UAV). The UAV was equipped with a multispectral sensor system providing the bands described in Table 2. After each capture flight, the objects were placed in a different environment of the same area and captured again, resulting in seven different locations for each target in both areas (the battery life of the UAV limited the number of capture flights per area to seven). When the first area had been captured seven times, the same process was repeated for the second area. The entire capture process was conducted on three different days in three different seasons: spring in May, summer in August, and autumn in November. This was done to provide variety in the data in order to make the results as meaningful as possible. Consequently, all camouflaged targets were captured in fourteen different environments (seven locations in two areas) in three different seasons, with only a few exceptions:

- The yellow 3D camouflage net was not used in area A in summer or autumn, as the environment was all green and no appropriate spot could be found for it.
- Only four capture flights over area B were conducted in summer, as the UAV broke during the experiments and could not be repaired in time.
- The yellow 3D camouflage net was left in the same place on all four summer capture flights in area B, as it had been overlooked when the camouflaged targets were rearranged.

**Figure 3:**
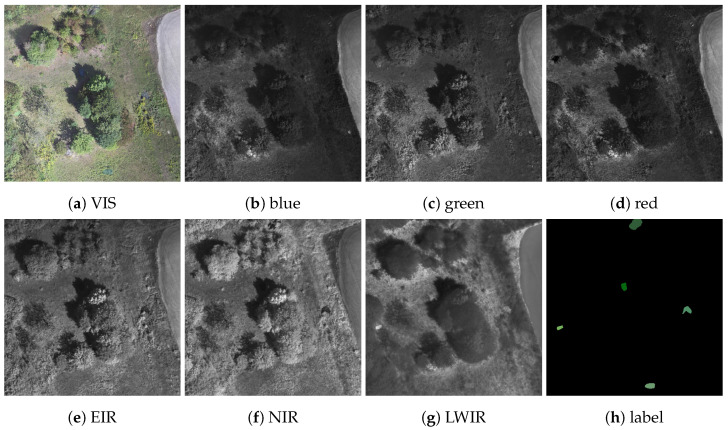
Sample capture of the dataset, with bands from VIS (**a**) to LWIR (**g**) and a ground truth mask (**h**) identifying all captured camouflaged targets. Note that each camouflaged target is denoted in a different color, making for five different targets in the scene.

The final dataset, called eXtended Multispectral Dataset for Camouflage Detection (MUDCAD-X), was not derived directly from the acquired data, instead being derived from orthophotos generated separately for each sensor band. Two of these orthophotos, computed from the visual band images, have already been shown in Figure 1. The orthophotos were generated with a ground sample distance (GSD) of 10$\frac{\mathrm{cm}}{\mathrm{px}}$ using the command line toolkit *Open Drone Map* [33]. Using a sliding window with a resolution of 512 by 512 pixels, the captures of the final dataset were cropped from the orthophotos to ensure that each capture contained at least a single camouflaged target. In addition, the individual sensor bands of each capture were pixel-aligned using Enhanced Correlation Coefficient Maximization [34] provided by the computer vision library *OpenCV* [35]. Figure 3 shows a sample capture of the dataset with all bands from VIS to LWIR (Figure 3a–g) and multiple different camouflaged targets in the scene that are identified by the ground truth mask in Figure 3h. In total, the final dataset contained 853 annotated and pixel aligned multispectral captures, each with a resolution of 512 by 512 pixels, a GSD of 10$\frac{\mathrm{cm}}{\mathrm{px}}$, and containing at least a single camouflaged target. The ground truth masks were created using the Computer Vision Annotation Tool v2.3.0 [36].

### 2.2. Measuring Sensor Performance

In order to train a machine learning model to predict the extent to which camouflaged targets are exposed in a given sensor band, a metric describing that extent is first required. Because the prediction is made for the entire sensor band as a single unit, this metric must consider the entire sensor band. For this purpose, in this paper we introduce the Target Visibility Index (TVI), provided in Equation (1), where $\mu_{T}$ is the mean over all pixel values belonging to the camouflaged target, $\mu_{B}$ is the mean over all pixel values belonging to the background, $\sigma_{T}$ is the standard deviation of all pixel values belonging to the camouflaged target, and $\sigma_{B}$ is the standard deviation of all pixel values belonging to the background. The mean and standard deviation are computationally efficient and commonly used statistical properties in well-established image metrics for a wide range of problems [31,32]. As such, they were employed for the TVI.

(1) $$\mathrm{TVI}={\left(\mu_{T}-\mu_{B}\right)}^{2}{\left(1-2\sigma_{T}\right)}^{2}{\left(1-2\sigma_{B}\right)}^{2}$$

**Figure 4:**
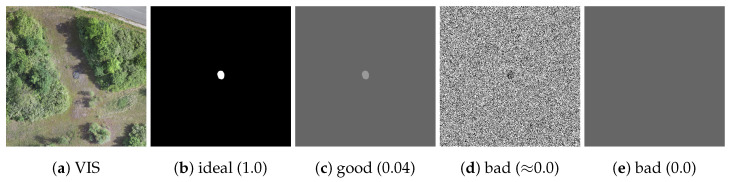
Demonstration of camouflaged target visibility, where (**b**) corresponds to an ideal band for the scene depicted in (**a**). Likewise, (**c**) corresponds to a band with good visibility of the target, while (**d**,**e**) correspond to a band with poor or no visibility of the target. The associated TVIs are shown in parentheses.

In general, the TVI is based on the idea that an ideal sensor band exposes a camouflaged target as much as possible, which is illustrated in Figure 4. The visual image in Figure 4a shows a scene containing a single camouflaged target, the green 3D camouflage net. According to the TVI, the corresponding ideal sensor band for that exact same scene is depicted in Figure 4b. All pixel values belonging to the camouflaged target differ as much as possible from all pixel values belonging to the background, resulting in the highest possible value of the TVI (1.0). The first factor of the TVI (${\left(\mu_{T}-\mu_{B}\right)}^{2}$) serves as an approximate measure of fulfillment of that property. It is zero when the difference between the camouflaged target pixel values and the background pixel values is zero and one when the difference between the camouflaged target pixel values and the background pixel values is maximal. Thus, it can be interpreted as a measure of contrast [30] between the target and the background. However, the difference between the mean values does not sufficiently describe the extent to which the target is exposed, as can be seen in Figure 4c,d. In both bands, the respective means over all pixel values belonging to the camouflaged target are identical, as are the respective means over all pixel values belonging to the background. Consequently, the first factor of the TVI (${\left(\mu_{T}-\mu_{B}\right)}^{2}$) yields the same result (0.04) in both cases. However, the difference in means in Figure 4d results from two different distributions of two pixel values, while in Figure 4c it results from a difference of two constant pixel values. Considering the exemplary case that the difference of mean values is already at its maximum, a band such as that in Figure 4c would most likely be preferable over the one in Figure 4d in an actual reconnaissance scenario. Therefore, the metric must take into account the distribution of the pixel values belonging to the background and the distribution of the pixel values belonging to the camouflaged target. To ensure that small spreads in the pixel value distributions are preferable over large spreads, the TVI implements the second (${\left(1-2\sigma_{T}\right)}^{2}$) and third (${\left(1-2\sigma_{B}\right)}^{2}$) factors, which penalize large spreads of pixel values and favor small spreads. In essence, the greater the TVI, the closer the camouflaged target pixel values and the background pixel values are to each other, respectively. Each factor equals zero if the spread of the respective pixel values is maximal and one if there is no spread at all. Because there is no spread in both distributions in Figure 4c, both factors are one and the first factor determines the final TVI. In contrast, the spread in both distributions in Figure 4d is close to the maximum, resulting in a TVI close to zero. However, there are limits, as shown in Figure 4e. Although the standard deviations for the target and background pixels are zero, their means are equal. As a result, the first factor of the TVI equals zero, leading to a TVI of zero as well. Eventually, the TVI can only be maximal when there is minimal spread in both camouflaged target pixel values and background pixel values, and when the difference between their mean values is maximal.

The TVI is designed for single-channel images and a range of values from zero to one. Other ranges must be normalized, or the TVI will produce inconclusive results. Each individual factor of the TVI ranges between zero and one. If one of the means is zero and the other is one, then the first factor of the TVI is one. If the means are equal, then the first factor is zero regardless of their actual values. Because the theoretical maximum of the standard deviation is 0.5 for a range of values from zero to one [37], the second and third factors of the TVI are zero for the maximum standard deviation and one for zero standard deviation. With all factors ranging between one and zero, the TVI range is between zero and one. The factors are multiplicatively combined to prevent a strong individual factor from outweighing a weak individual factor, which would be possible in an additive combination, for instance. Additionally, each factor is squared to avoid negative values and retain the differentiability of the TVI, which might be useful if the TVI is used for numerical optimization problems.

**Figure 5:**
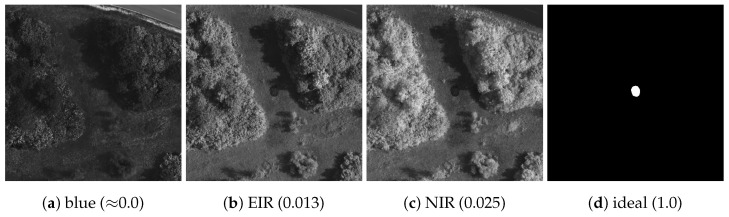
Demonstration of the Target Visibility Index (TVI), producing relatively low values for bad visibility of the target in (**a**) and relatively high values for good target visibility in (**b**,**c**). The ideal band in (**d**) results in a TVI of 1.

Figure 5 shows real-world examples of the TVI. As can be observed, the TVI is very low for the blue band (Figure 5a) and relatively high for the EIR band (Figure 5b) and NIR band (Figure 5c). This is consistent with the expected behavior of the metric, as the target appears to be much more exposed in the EIR and NIR bands than in the blue band. It can be seen that the TVI generally produces relatively low values for real-world images, even when the camouflaged target is easily distinguishable from its surroundings. At this point, it is important to note that the design of the TVI is based on those edge cases where it is equal to either one, as shown in Figure 4b, or zero, as shown in Figure 4d,e. The closer the sensor band is to one of these edge cases, the closer the TVI is to zero or one, where *closer* is mathematically defined by the factors provided in Equation (1). For any TVI value in between these edge cases, its true meaning in terms of target visibility is difficult to determine and does not necessarily correspond to human perception. For example, if the target in Figure 5c were placed in the shadows of the tree line immediately next to it, it would be much less visible to the human eye; however, in terms of the TVI, the visibility of the target would be roughly the same in both cases, as the change in the mean and standard deviation of the background pixels would be negligible. Notably, actual human perception of visibility is currently the subject of active research, which is beyond the scope of this work except for those trivial edge cases in which the TVI generates predefined values of zero and one. The TVI quantifies target visibility as a single value in a computationally efficient and comparable way, which naturally involves approximation; ultimately, this is necessary in order to measure and compare the extent to which a target is exposed in different sensor bands.

### 2.3. Predicting Sensor Performance

**Figure 6:**
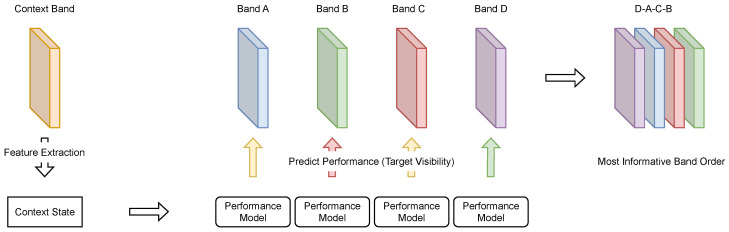
Conceptual basis of sensor performance prediction (target visibility). First, the context state is extracted by image descriptors from a preselected context band. Based on the context state, each performance model then predicts the target visibility for its associated band. The bands can be sorted after the predictions are made, allowing them to be ordered from the most informative to the least informative band. The green, yellow, and red prediction arrows indicate good, medium, and bad performance, respectively.

To predict sensor performance, in this paper we introduce the concept illustrated in Figure 6. Considering a multispectral sensor system with multiple different bands, a preselected context band is used to extract a context state that provides abstract information about the current environment and scenery. From the context state, the individual performance models predict the performance of their associated sensor bands. In the illustrated example, the predicted performance is high for band D, medium for bands A and C, and low for band B. Finally, the sensor bands are ranked by their performance predictions in order to obtain the subset of sensor bands that is most likely to provide the highest visibility of camouflaged targets. This greatly reduces the amount of information that must be processed in any subsequent evaluation instance.

In this work, the context state is extracted from the gray-level converted visual band using 16 bit rotation-invariant uniform local binary patterns ($LBP_{16}^{riu2}$ operator) [38] and the fourteen Haralick features [39]. Both are computationally inexpensive and common choices for feature extractors in image classification problems [40,41,42], where an abstract representation of the scene to be classified is required as well. Table 3 shows the final composition of the context state. The LBPs were extracted with a radius of 2 px and a resolution of 16 using the implementation of the ImageFeatures.jl package of the Julia Programming Language [43]. To obtain the first eighteen values of the context state, the histogram over the extracted rotation-invariant uniform patterns was computed. Each value of the histogram represents the number of occurrences of each individual pattern. Because there are exactly seventeen rotation-invariant uniform patterns for a bit size of 16, the resulting histogram holds eighteen values, where the last one accounts for the occurrences of all non-uniform patterns. In the last step, the histogram is normalized to ensure that the values of the histogram sum to one. The remaining Haralick features of the context state were computed using the Python package Mahotas [44]. Each value of the first fourteen Haralick features is an average of four individual features values produced by four different gray-level co-occurrence matrices, each generated for a radius of 1 px and the directions left, right, up, and down. The second fourteen Haralick features contain the differences between the maximum and minimum values generated by each of the four individual gray-level co-occurrence matrices. In total, the context state consists of 46 features that abstractly describe the environmental situation based on the preselected context band.

For the performance models that predict the sensor band performance from the context state, three machine learning methods for regression tasks were applied: ϵ-Support Vector Regression (ϵ-SVR), Random Forests (RFs), and Gradient Boosted Trees (GBTs). All are based on different concepts, have been thoroughly studied, and are commonly used for complex regression tasks. In addition, their training is efficient and a robust implementation is usually available for the most popular programming languages. Therefore, they were chosen for the regression task in this work. The ϵ-SVR, RFs, and GBTs were trained and evaluated using the common interface of the Machine Learning Framework for Julia [45], where the models relied on the LIBSVM [46], DecisionTree.jl [47], and XGBoost [48] backends, respectively. The parameter selections used to train the models are introduced in Section 3.1.

## 3. Experiments and Results

This section first introduces the parameters and data used to train the machine learning models (i.e., performance models) in Section 3.1. Afterwards, the evaluation of the prediction performances of all models is presented in Section 3.2.

### 3.1. Training

In order to divide the dataset introduced in Section 2.1 into training and test data, 80% of the captures were randomly selected as training data and the remaining 20% were used to evaluate the models. In each capture, for each band except VIS the context state was extracted from the gray-level converted visual band as described in Section 2.3 and mapped to a single TVI. Because there are six bands (blue, green, red, EIR, NIR and LWIR), each context state maps to six different TVIs per capture. In the case of multiple camouflaged targets in the capture, the resulting TVIs had to be reduced to a single value. This was achieved by averaging all of the individual TVIs calculated separately for each camouflaged target. Thus, a camouflaged target belongs to the background of every other camouflaged target in the scene. Although averaging could dilute the mappings from the context state to the TVI, it is able to consider all targets in the scene equally for the single sensor performance value. The context states were additionally z-normalized, leading to a mean value of zero and a standard deviation of one for each feature value over all context states. The means and standard deviations required for the normalization were calculated for the training data only, then applied to both the training and test data.

Considering a reconnaissance scenario in which a priori knowledge on the camouflaged targets is available, it could be beneficial to employ performance models that are able to account for this additional knowledge. For example, if the camouflaged targets are known to be located in green environments such as woods, bushes, and grass, a performance model trained only on targets commonly used in these environments may outperform a model trained on additional kinds of targets. Therefore, the models were additionally trained on data for which the resulting TVI for each sensor band was not calculated over all camouflaged targets in the scene, only over those belonging to specific target group, as has been already introduced above in Table 1. This reduces the potential dilution caused by averaging over the TVIs of targets in different target groups. Because not every capture in the dataset contains a camouflaged target of each target group, the training and testing splits and feature normalization for the specialized models were performed only on the number of captures that actually contained a target of the respective target group. With three different target groups, the models were trained on a total of four different data variations: one for each of the target groups, and one in which the target groups were ignored. With six bands for each capture, three different machine learning models, and four different data variations, a total of 72 models were trained. After training, the models use a normalized feature vector extracted from a gray-level converted visual band to predict the TVI for their associated sensor band. For the models trained on data where the TVI was calculated only for a specific target group, their predictions consider only the targets belonging to that specific target group. In contrast, the predictions of the models trained on data containing all camouflaged targets consider all target groups.

The optimal parameters for each model were searched by a simple grid scan based on cross-validation over the training data with five folds and utilizing the root mean square error (RSME). The four-part Table 4 shows the final training parameter configurations for the models considering all target groups, only targets belonging to the green target group, only targets belonging to the gray target group, and only targets belonging to the yellow target group, respectively. Note that the predictions of the individual models were not evaluated further; instead, the evaluation of the models is based on comparisons of the predicted most informative band orders with the actual most informative band orders, which is explained in detail in Section 3.2.

### 3.2. Evaluation

With the models were already trained on 80% randomly selected data, their evaluation was performed on the remaining 20%. For each capture of the test data, the models had to predict the TVI for their associated sensor band. Afterwards, the bands were sorted from the band with the highest TVI prediction to the band with the lowest TVI prediction. The result of this sorting is called the predicted most informative band order. Because the targets in the test data were known, the actual TVI for each sensor band could be calculated, as was done for the training data during the training procedure. From the calculated TVIs for each sensor band, the bands were then sorted from the band with the highest calculated TVI to the band with the lowest calculated TVI. The result of this sorting is called the actual most informative band order. With the actual and predicted most informative band for each capture in the dataset, the band orders could then be compared for accuracy. For example, the Top-1 accuracy is the proportion of captures in the test data where the first band of the predicted most informative band order is the same as the first band of the actual most informative band order. The same principle applies to the Top-3 accuracy, which is the proportion of captures in the test data in which the first band of the predicted most informative band order is one of the first three bands of the actual most informative band order.

Tp compare the predicted most informative band orders generated by the performance models using a static approach, a static baseline was introduced. The static baseline provides only the single most informative band order over all captures (the static most informative band order). It is motivated by the idea that it is not worth utilizing performance models if a simple static most informative band order already performs better than the predicted most informative band orders over all captures in the test data. The static most informative band order was obtained by penalizing each band using its position in the actual most informative band order over all captures in the training data. For example, as there are six bands, the most informative band is penalized by one and the least informative band is penalized by six. By accumulating the penalties of each band over all captures, the bands can be sorted from the band with the lowest accumulated penalty to the band with the highest accumulated penalty. This sorting results in the static most informative band order. Because the models were trained on four different sets of training data (one with all target groups, one with only green targets, one with only gray targets, and one with only yellow targets), there are four separate static most informative band orders:

- LWIR, NIR, EIR, red, blue, and green for any camouflaged target.
- NIR, LWIR, EIR, green, blue, and red for green camouflaged targets.
- NIR, blue, EIR, red, green, and LWIR for gray camouflaged targets.
- Red, blue, LWIR, green, EIR, and NIR for yellow camouflaged targets.

Table 5 shows the prediction accuracies of each model as a percentage. The individual quarters of the table contain the results of the general models trained on any camouflaged target and the specialized models trained only on green, gray, and yellow camouflaged targets, respectively. Each individual quarter provides four tables showing the prediction accuracies of the different machine learning models along with the prediction accuracies of the static baselines. Here, each cell contains the proportion of captures in the test data where the <row number> predicted most informative bands were among the <column number> actual most informative bands. For example, the value of the second column in the first row is the proportion of captures in which the first band of the predicted most informative band order was among the first two bands of the actual most informative band order (Top-2 accuracy). Likewise, the value of the third column and the second row represents the proportion of captures in which the first two bands of the predicted most informative band order were among the first three bands of the actual most informative band order. Therefore, the value of the first column and first row stands for the proportion in which the predicted most informative band was the actual most informative band (Top-1 accuracy). Although there are six different bands, the individual tables consist of only five columns and rows. This is for better clarity, as the last column would contain ones for each model and for the baseline. In all of the results for the different training procedures, the best predictions for each accuracy category are shown in bold.

As can be seen for the general models, the ϵ-SVR and Random Forest models are superior to the XGBoost models in terms of prediction accuracy. The ϵ-SVR models provide slightly higher prediction accuracy than the Random Forest models, with eight top results compared to six top results. While the XGBoost model achieves only two top results, the static baseline is inferior to all machine learning models, without a single top result. In addition, all models reach over 50% Top-1 accuracy and over 80% Top-3 accuracy. This means that the predicted most informative band is the actual most informative band more than half of the time, while most of the time the predicted most informative band is at least one of the three actual most informative bands. Similar results are shown by the models specializing in targets belonging to the green target group. However, the ϵ-SVR model significantly outperforms all other models, achieving ten of the top fifteen results. In addition, the prediction accuracies are generally slightly higher than for the general case. The same applies to the results of the specialized gray target models, where only the number of top results is evenly split between the ϵ-SVR and the Random Forest models. In contrast, the specialized yellow target models are inferior to the static baseline in terms of the number of best results. Nonetheless, the first row, which represents the prediction accuracy of the single most informative band, is dominated by the top results of the ϵ-SVR model. Overall, the ϵ-SVR models perform the best, with the Random Forest and XGBoost models performing only slightly worse.

To highlight the effectiveness of the performance models, Table 6 shows the relative accuracy improvements of each model compared to the static baseline, for which the prediction results are shown in the lower right of each quarter in Table 5. Apart from this, Table 6 has the same structure as Table 5. As can be seen, the general models achieve significant improvements over the static baseline, peaking at 45.2%, 35.7%, and 22.5% for the ϵ-SVR, Random Forest, and XGBoost models, respectively. Although the XGBoost model is slightly worse in one of the accuracy categories, it generally achieves much higher accuracy than the static baseline. Nonetheless, its improvements are not as great as those of the ϵ-SVR and Random Forest models. The green target models achieve similar, though slightly lower overall improvements over the static baseline, with a notable very high increase in Top-1 accuracy. Likewise, the gray target models significantly outperform the static baseline, especially in Top-1 accuracy, where the prediction accuracy is more than doubled with improvements of up to 140%. Despite strong improvements in Top-1 accuracy, the yellow target models fail to improve in many of the accuracy categories. However, as noted above, the single most informative band is predicted the best by the ϵ-SVR model. In general, when considering both general and specialized models, all of the models are superior to the static baseline.

Table 7 shows the benefits of the performance models trained only on camouflaged targets belonging to a specific target group. Again, the structure of the table is the same as Table 5. The cells show the relative improvements in each accuracy category of the specialized models compared to the general models for only that target group on which the models were specialized. For example, the improvement of the green target models was obtained by comparing their prediction accuracy with that of the general models, while, the prediction accuracy of the general models was obtained by considering only green targets rather than of all targets. The same approach was applied to obtain the improvements of the gray and yellow target models. In this way, the benefits of the specialized models were quantified in an objective manner. As can be seen for the green target models, specialization leads to an overall improvement in prediction accuracy. The ϵ-SVR model achieves the most significant improvements, peaking at nearly three times the accuracy, a 191.9% improvement. The improvements are even greater for the gray target models, with the Random Forest model achieving nine top improvements and a 270% increase in Top-1 accuracy. Similar improvements can be observed for the yellow target models, with the XGBoost model showing the greatest improvements (a maximum increase in accuracy of 767.4%). Overall, the specialized models clearly outperform the general models within their respective target groups.

## 4. Discussion

This section first discusses the possible implications of the proposed sensor prediction approach in Section 4.1, followed by a discussion of its limitations in Section 4.2. Finally, future research prospects are reviewed in Section 4.3.

### 4.1. Implications

Altogether, our results demonstrate the effectiveness of the sensor performance prediction approach presented in this paper, with the ϵ-SVR models showing the most robust performance. While not perfect, the performance models were able to learn a meaningful relationship between the context state and the corresponding TVI, which supports the utility of the extracted features and the expressiveness of the TVI. In the general case, when only the three predicted most informative bands were considered, the actual most informative band was most likely among them (around 84%), while the associated workload was reduced by a half compared to processing all six bands. Although a reduction from six to three sensor bands may seem small in absolute terms, the sensor performance prediction approach is adaptable to any number of bands. For the sake of simplicity and clarity, however, our evaluation of the proposed performance prediction approach focuses on the raw bands of the multispectral sensor system employed in this study. While not explicitly explored here, the nature of the proposed methodology suggests similar results for a smaller or larger number of bands. Therefore, the proposed method could significantly increase the utility of multispectral sensor systems in real-world applications. For example, reconnaissance drones could be equipped with much more powerful multispectral sensor systems, as the increased number of sensor bands would not result in an equally increased workload. In this case, the sensor performance prediction approach would determine the most informative bands and ignore the least informative bands. The resulting increased meaningfulness of each sensor band and the additional spectral information due to the larger number of bands could greatly improve camouflage detection performance in reconnaissance scenarios.

In addition, our evaluation shows that specializing the performance models for certain target groups can significantly increase prediction performance. This could potentially increase reconnaissance performance for scenarios in which camouflaged targets are known to be present in a specific kind of environment, as the specialized models are able to focus on the environment associated with their specific target group even when the reconnaissance area consists of different kinds of environments. In contrast, the general models consider all relevant environments even when camouflaged targets are known to be present in only one environment. Thus, the predictions of the specialized models are more closely tailored to the environment in which the camouflaged targets are located, resulting in greater camouflage detection performance. Naturally, the specialized models cannot generalize to environments that are not associated with their specific target group. For this reason, they can only be of use if this specific kind of prior knowledge is available.

Comparing the performance models to a static baseline further highlights the benefits of their application. Although the use of static most informative band orders is computationally less expensive than the use of performance models, the former approach is not able to achieve the same level of prediction accuracy. Therefore, the comparatively low computational overhead of the performance models is preferable to the lower performance of the static baselines. However, it should be noted that the yellow target models did not significantly outperform their associated static baselines. This could be due to the relatively small amount of training data, as yellow targets were not as common as green targets in our dataset. A lack of training data may have prevented the performance models from sufficiently learning the complex relationship between the context state and the TVI, resulting in more limited generalization capabilities. On the other hand, even though the dominance of gray targets in the dataset was even lower than that of yellow targets, the performance models for gray targets were far superior to the static baseline. This could be due to the relationship between the context state and the TVI being less complex for the gray target models than for the yellow target models. Unfortunately, the causes of the relatively poor performance of the yellow target models could not be further explored in this study.

Because the idea behind the performance prediction approach is not strictly bound to the camouflage detection task, it can be generalized and applied to other multispectral sensing problems. In the present work, sensor performance corresponds to the TVI; however, this particular metric could be replaced with any other metric that fits the problem at hand. For example, such a metric could describe the ability of a sensor band to detect invasive species. In this case, the performance models simply had to learn the relationship between the context state and the new metric instead of the TVI. Even the context state is not specifically tailored to camouflage detection, as it is generated by general image descriptors. Therefore, it could be of equal utility in other use cases. Considering the positive results we obtained when applying the proposed concept to multispectral camouflage detection, it could be equally successful when applied to other tasks.

### 4.2. Limitations

Although the value of the proposed performance prediction approach has been confirmed, it should be noted that all of our results are based on the Target Visibility Index metric introduced in this paper. As has already been discussed in Section 2.2, the TVI defines visibility using its mathematical formula, which does not necessarily correspond to human perception. Therefore, certain targets that may actually have poor visibility to the human eye can result in a relatively high TVI, and vice versa. This behavior may have led to predictions of the performance models that were correct with respect to the TVI and incorrect with respect to the human eye. As a result, the benefits of the proposed performance prediction approach may be limited in a real-world application involving humans.

Furthermore, because each camouflaged target possesses unique spectral characteristics, the mappings from the context state to the TVI may have been diluted in the data. This may have limited the achieved prediction accuracy of the performance models. For example, while a target in one environment will result in a different TVI than another target in the same environment, the context state will not have changed in either case, as the context state is mainly determined by the scenery and not by the targets. This leads to mappings from one context state being applied to different TVIs for the same sensor band, which could have confused the training of the performance models. The averaging of all TVIs in the same capture could have further amplified this potential issue, as already mentioned in Section 3.1. However, the environment, and consequently the context state, already provide an indication of the properties of the camouflaged targets, as green targets, for example, are usually found in green environments. Therefore, the TVI may follow a certain distribution for a given environment, which could have limited the potential negative effects on the training of the performance models.

In addition, it is important to note that the performance models were not evaluated for their ability to generalize to unknown camouflaged targets. Although the data were split into training and test data, all of the camouflaged targets were part of both datasets. However, the test data contained captures that were completely unknown to the performance models, on which they showed high prediction accuracy. This suggests that the proposed sensor prediction approach has great potential for generalization.

Another limiting factor on the prediction accuracy could have been the meaningfulness of the context state extracted from the visual band. Because the context state results from relatively simple feature extractors, the performance models may not have learned every aspect of the complex relationship between the environmental situation and the TVI. More sophisticated and computationally expensive feature extraction methods, such as convolutional neural networks, might have provided even more meaningful context states. With more information about the environmental situation available in the context state, the performance models may have achieved even higher prediction accuracy. However, the computational resources on a small reconnaissance drone in a real-world application are usually limited. This requires computationally inexpensive methods for both the feature extraction process and the performance models, which have been successfully implemented and demonstrated in this paper.

### 4.3. Future Research

Our future research will primarily focus on predicting sensor performance for multispectral sensor systems with an even higher number of bands. In addition, the sensor performance prediction approach proposed in this paper will be included in a larger framework in which the most informative bands will be incorporated into a computer-aided camouflage detection system. As noted above, richer features in the context state may improve the prediction accuracy of the performance models, which will be another subject of future research.

## 5. Conclusions

The sensor performance prediction approach presented in this paper has been shown to be a successful method for obtaining those sensor bands that best expose camouflaged targets. This increases the meaningfulness of each individual sensor band, allowing for the use of more powerful multispectral sensor systems. As a result, camouflage detection performance may be significantly increased in real-world reconnaissance scenarios.

In addition, specialized training of the performance models showed promising improvements in prediction accuracy. This may further increase camouflage detection performance in real-world reconnaissance scenarios, provided that the necessary prior knowledge of the camouflaged targets to be exposed is available.

Moreover, it has been shown that the performance models are superior to the statically computed most informative band order. This indicates the existence of a complex relationship between the environmental situation and the TVI that can be successfully exploited and learned by performance models. Therefore, the benefits of the proposed performance prediction approach outweigh its computational overhead compared to a static baseline and motivate its application in real-world reconnaissance scenarios.

However, it should be noted that all results are based on the TVI, which is an experimental metric of sensor performance in the context of camouflaged target detection. Because the TVI does not necessarily correspond to human perception and is difficult to apply to multiple targets in the same scene, the range of applications of the proposed sensor performance prediction approach may be limited. In addition, the context state may not be as informative as it might have been with more sophisticated feature extraction methods, which in turn may have limited the prediction accuracy of the performance models.

Future research will address the integration of the proposed sensor prediction approach into an automated camouflaged target detection system and the generation of a richer context state.

## Figures and Tables

**Table 1:** All thirteen camouflaged targets and their corresponding target group captured in the dataset. The percentages show the proportion of each target or target group among the annotations in the dataset.

Camouflaged Target		Group
artificial turf	9.3%	green 65.8%
artificial hedge	9.4%	
green tarp	9.2%	
green 2D camouflage net	9.9%	
green 3D camouflage net	9.6%	
2 persons in green uniforms	18.4%	
gray tarp	3.1%	gray 11.4%
anthracite fleece	2.2%	
gray 3D camouflage net	6.2%	
yellow 3D camouflage net	5.9%	yellow 22.8%
2 persons in yellow uniforms	16.9%	

**Table 2:** The bands and their associated properties provided by each capture of the dataset.

Band	Center Wavelength	Bandwidth
visual (VIS)	-	-
blue	475 nm	32 nm
green	560 nm	27 nm
red	668 nm	14 nm
edge-infrared (EIR)	717 nm	12 nm
near-infrared (NIR)	842 nm	57 nm
long-wave infrared (LWIR)	10.5 μm	6 μm

**Table 3:** The structure of the context state extracted from the context band (VIS) using local binary patterns and Haralick features. The numbers correspond to the feature value positions of the context state.

LBP		Haralick	
uniform	non-uniform	mean	min-max
1–17	18	19–32	33–46

**Table 4:** The final training parameter configurations for each machine learning model (i.e., performance model), including the models that considered targets of any target group and the models that considering only targets belonging to one of the green, gray, or yellow target group. Unmentioned parameters were retained at the default values provided by their respective implementations. The trees, leaves, split, features, and fraction parameters of the Random Forest model specify the number of trees, minimum number of samples belonging to a single leaf node, minimum number of samples required for further splitting, number of random subfeatures for each tree, and fraction of random training samples for each tree, respectively. The rounds and depth parameters of the XGBoost model represent the maximum depth of each tree and the number of boosting rounds, respectively.

	ϵ-SVR		Random Forest					XGBoost			
	ϵ	C	Trees	Leaves	Split	Features	Fraction	Rounds	η	Depth	λ
any target models											
blue	0.00412	0.044	46	3	15	$\sqrt{46}$	1.0	200	0.05	6	2.5
green	0.00162	0.06	46	3	7	$\sqrt{46}$	1.0	400	0.025	2	0.001
red	0.0041	0.041	28	2	16	$\sqrt{46}$	0.8	450	0.1	2	30
EIR	0.0018	0.022	14	1	8	$\sqrt{46}$	0.9	275	0.04	6	0.1
NIR	0.00112	0.026	28	1	4	$\sqrt{46}$	1.0	125	0.05	4	0.001
LWIR	0.00747	0.1	23	1	3	$\sqrt{46}$	0.8	475	0.1	2	25
green target models											
blue	0.00068	0.038	41	3	5	$\sqrt{46}$	1.0	125	0.065	4	0.1
green	0.00202	0.038	14	1	5	$\sqrt{46}$	0.8	100	0.075	4	0.25
red	0.00163	0.1	28	1	2	46	1.0	150	0.06	4	0.001
EIR	0.00538	0.041	23	2	16	$\sqrt{46}$	0.6	75	0.08	2	0.001
NIR	0.00748	0.014	10	13	19	46	0.5	75	0.095	2	0.001
LWIR	0.00835	0.089	10	4	11	$\sqrt{46}$	0.8	300	0.1	2	27.5
gray target models											
blue	0.01	0.093	32	5	8	46	0.9	200	0.1	2	1.0
green	0.00689	0.03	23	4	6	$\sqrt{46}$	1.0	150	0.05	4	0.5
red	0.00996	0.018	19	11	2	46	0.5	100	0.095	2	50
EIR	0.00989	0.086	23	2	2	46	0.6	100	0.06	2	5.0
NIR	0.00985	0.028	19	9	11	$\sqrt{46}$	0.9	125	0.045	2	0.5
LWIR	0.00428	0.021	10	14	7	46	0.5	375	0.1	2	27.5
yellow target models											
blue	0.00144	0.08	14	5	9	$\sqrt{46}$	1.0	100	0.1	6	0.1
green	0.00705	0.081	14	4	14	$\sqrt{46}$	0.5	225	0.075	8	0.25
red	0.00847	0.096	41	3	2	$\sqrt{46}$	0.8	75	0.095	6	0.1
EIR	0.00299	0.006	10	1	7	46	1.0	125	0.06	4	0.1
NIR	0.00138	0.001	28	2	2	$\sqrt{46}$	0.6	125	0.095	2	50
LWIR	0.00668	0.1	37	4	12	46	1.0	200	0.09	12	0.1

**Table 5:** The prediction accuracies of all models (in percentages). The upper left quarter contains the results of the general models, while the others contain the results of the specialized models. Each individual table shows the prediction accuracy of the respective model, where first row of the table corresponds to the Top-1 accuracy in the first column and the Top-5 accuracy in last column. In the second row, the value in the second column represents the accuracy of predicting the two most informative bands, regardless of their order. Similarly, the third column represents the accuracy of predicting two bands out of the three actual most informative bands. The same pattern applies to all other cells as well. The best results within each target group are shown in bold.

Any Target Models										Green Target Models									
ϵ-SVR					Random Forest					ϵ-SVR					Random Forest				
**56.1**	**71.9**	83.6	88.9	95.3	51.5	67.8	**84.2**	88.3	97.1	57.3	**74.5**	86.0	**93.0**	**98.7**	58.0	72.6	84.7	91.7	97.5
	**35.7**	59.1	**71.3**	**88.9**		33.3	**59.6**	70.8	87.7		**42.7**	**73.2**	**84.7**	**93.0**		36.9	67.5	79.6	91.1
		**37.4**	**53.8**	72.5			35.7	53.2	**75.4**			47.8	68.2	**86.0**			49.7	**69.4**	84.7
			**23.4**	55.0				22.2	**59.6**				**47.1**	**71.3**				38.9	68.8
				34.5					**35.7**					38.9					**42.0**
XGBoost					Baseline					XGBoost					Baseline				
50.9	68.4	83.6	**92.4**	**97.7**	47.4	56.1	69.6	75.4	82.5	**58.6**	70.1	**86.6**	91.7	96.8	31.2	68.2	81.5	89.2	96.8
	29.8	57.9	70.8	87.1		24.6	48.0	57.9	71.9		35.7	68.8	82.8	90.4		28.7	60.5	73.2	83.4
		31.0	46.8	68.4			33.9	46.2	60.2			**51.0**	68.8	83.4			47.8	65.0	80.3
			18.1	53.8				19.9	48.5				44.6	65.6				35.0	54.1
				34.5					28.7					39.5					25.5
**Gray Target Models**										**Yellow Target Models**									
ϵ-SVR					Random Forest					ϵ-SVR					Random Forest				
57.4	**77.0**	**86.9**	**93.4**	**98.4**	**59.0**	**77.0**	**86.9**	91.8	**98.4**	**49.5**	**75.7**	91.3	**95.1**	99.0	47.6	74.8	85.4	94.2	**100.0**
	34.4	59.0	**82.0**	**93.4**		**44.3**	**72.1**	77.0	90.2		35.9	66.0	82.5	93.2		**37.9**	66.0	83.5	93.2
		24.6	57.4	72.1			29.5	52.5	78.7			39.8	**74.8**	90.3			34.0	72.8	90.3
			**34.4**	62.3				24.6	**63.9**				51.5	**84.5**				54.4	80.6
				29.5					34.4					74.8					72.8
XGBoost					Baseline					XGBoost					Baseline				
50.8	**77.0**	85.2	88.5	96.7	24.6	63.9	83.6	91.8	98.4	43.7	69.9	83.5	91.3	99.0	31.1	71.8	**92.2**	94.2	96.1
	36.1	65.6	77.0	88.5		24.6	44.3	70.5	88.5		36.9	69.9	84.5	**96.1**		31.1	**75.7**	**89.3**	95.1
		**34.4**	**60.7**	**82.0**			18.0	41.0	59.0			**45.6**	71.8	90.3			38.8	65.0	**92.2**
			26.2	62.3				24.6	47.5				**57.3**	**84.5**				53.4	**84.5**
				**39.3**					34.4					74.8					**75.7**

**Table 6:** The relative prediction accuracy improvements in each category of all models compared to their respective statically computed baselines (in percentages). The best results are shown in bold, even if all models predicted worse than the static baseline. This table follows the same structure as Table 5.

Any Target Models										Green Target Models									
ϵ-SVR					Random Forest					ϵ-SVR					Random Forest				
**18.5**	**28.1**	20.2	17.8	15.6	8.6	20.8	**21.0**	17.1	17.7	83.7	**9.3**	5.5	**4.3**	**2.0**	85.7	6.5	3.9	2.9	0.7
	**45.2**	23.2	**23.2**	**23.6**		35.7	**24.4**	22.2	22.0		**48.9**	**21.1**	**15.7**	**11.5**		28.9	11.6	8.7	9.2
		**10.3**	**16.5**	20.4			5.2	15.2	**25.2**			0.0	4.9	**7.1**			4.0	**6.9**	5.6
			**17.6**	13.3				11.8	**22.9**				**34.5**	**31.8**				10.9	27.1
				20.4					**24.5**					52.5					**65.0**
XGBoost										XGBoost									
7.4	21.9	20.2	**22.5**	**18.4**						**87.8**	2.8	**6.3**	2.9	0.0					
	21.4	20.7	22.2	21.1							24.4	13.7	13.0	8.4					
		−8.6	1.3	13.6								**6.7**	5.9	4.0					
			−8.8	10.8									27.3	21.2					
				20.4										55.0					
**Gray Target Models**										**Yellow Target Models**									
ϵ-SVR					Random Forest					ϵ-SVR					Random Forest				
133.3	**20.5**	**3.9**	**1.8**	**0.0**	**140.0**	**20.5**	**3.9**	0.0	**0.0**	**59.4**	**5.4**	**−1.1**	**1.0**	3.0	53.1	4.1	−7.4	0.0	**4.0**
	40.0	33.3	**16.3**	**5.6**		**80.0**	**63.0**	9.3	1.9		15.6	−12.8	−7.6	−2.0		**21.9**	−12.8	−6.5	−2.0
		36.4	40.0	22.2			63.6	28.0	33.3			2.5	**14.9**	**−2.1**			−12.5	11.9	**−2.1**
			**40.0**	31.0				0.0	**34.5**				−3.6	**0.0**				1.8	−4.6
				−14.3					0.0					**−1.3**					−3.8
XGBoost										XGBoost									
106.7	**20.5**	2.0	−3.6	−1.7						40.6	−2.7	−9.5	−3.1	3.0					
	46.7	48.1	9.3	0.0							18.8	**−7.7**	**−5.4**	**1.0**					
		**90.9**	**48.0**	**38.9**								**17.5**	10.4	**−2.1**					
			6.7	31.0									**7.3**	**0.0**					
				**14.3**										**−1.3**					

**Table 7:** The relative prediction accuracy improvements of the specialized models compared to the general models. Note that the improvements are based on the prediction accuracy of the general models that results when considering only those targets considered for the prediction accuracy of the respective specialized model, and not on the prediction accuracy of the general models shown in Table 5. This table follows the same structure as Table 5.

										Green Target Models									
										ϵ-SVR					Random Forest				
										−0.8	**1.7**	1.8	3.3	**3.9**	4.9	−0.9	1.0	**4.0**	2.5
											**22.7**	**35.6**	**20.7**	9.3		8.1	23.5	14.5	**9.4**
												47.9	**35.5**	**28.2**			50.9	34.7	20.7
													**191.9**	**57.3**				172.0	38.4
														**69.1**					50.4
										XGBoost									
										**7.2**	−3.6	**2.5**	2.5	3.2					
											4.4	21.7	14.9	7.9					
												**54.8**	33.4	16.8					
													111.1	32.0					
														44.5					
**Gray Target Models**										**Yellow Target Models**									
ϵ-SVR					Random Forest					ϵ-SVR					Random Forest				
182.8	77.2	53.7	40.2	**19.1**	**270.2**	**112.7**	**71.3**	**54.5**	**19.1**	38.1	63.8	**72.7**	**36.3**	**19.3**	**48.3**	**65.1**	48.5	21.7	10.4
	13.1	40.4	**66.3**	21.7		**45.4**	**55.5**	47.7	**24.4**		246.2	133.3	98.8	**62.0**		301.4	191.6	96.7	47.5
		−0.2	80.0	42.2			45.4	72.4	**59.7**			122.1	147.6	108.1			260.2	175.7	117.5
			**375.1**	**79.1**				112.1	76.5				319.6	198.4				343.3	205.1
				85.1					137.5					366.1					328.8
XGBoost										XGBoost									
150.5	96.9	59.0	35.7	15.1						25.2	57.7	50.0	19.4	16.6					
	38.3	46.0	51.9	17.5							**334.5**	**208.7**	**108.2**	61.7					
		**58.4**	**109.3**	52.9								**706.1**	**231.1**	**139.3**					
			158.5	71.9									**767.4**	**258.1**					
				**146.8**										**560.4**					

## Data Availability

The eXtended Multispectral Dataset for Camouflage Detection (MUDCAD-X) dataset is publicly available on GitHub: https://github.com/Tobias-UniBwM/MUDCAD-X (accessed on 11 August 2023).

## Abbreviations

The following abbreviations are used in this manuscript:

EIR	Edge Infra-red/Red-edge
GBT	Gradient Boosted Tree
LBP	Local Binary Pattern
LWIR	Long-Wave Infra-Red
MUDCAD-X	eXtendend Multispectral Dataset for Camouflage Detection
NDRE	Normalized Difference Red-Edge index
NDVI	Normalized Difference Vegetation Index
NIR	Near Infra-Red
RF	Random Forest
RMSE	Root Mean Square Error
TVI	Target Visibility Index
UAV	Unmanned Aerial Vehicle
UniBwM	University of the Bundeswehr Munich
VIS	Visual
XGBoost	eXtreme Gradient Boosting
